# Disparity in access to orthopedic surgery between public and private healthcare insurance: a nationwide population-based study

**DOI:** 10.1186/s12891-025-08295-7

**Published:** 2025-05-10

**Authors:** María Jesús Lira, Paula Pino, Catalina Vidal, Pamela Mery, Sebastián Irarrázaval, Jaime Cerda, Jorge Vergara

**Affiliations:** 1 Department of Orthopedic Surgery, Pontificia Universidad Católica de Chile, Diagonal Paraguay Nº 362, 3rd floor, Santiago de, Chile; 2 Public Health Department, Pontificia Universidad Católica de Chile, Santiago de, Chile

**Keywords:** Disparity, Orthopedic surgery, Healthcare insurance

## Abstract

**Background:**

This study aimed to evaluate if access to orthopedic surgery differs by healthcare coverage in a country with a dual healthcare system adjusted by age, sex, and urgent and elective conditions. We hypothesize that differential access would exist according to the type of healthcare coverage. This difference would accentuate when analyzing access to elective orthopedic surgery.

**Methods:**

A cross-sectional, population-based design was used to investigate orthopedic surgery rates in Chile in 2018. The rates of orthopedic surgeries provided under the private and public healthcare systems were calculated per 1,000 inhabitants based on data collected from the Hospital Discharge Registry provided by the Chilean Ministry of Health. ICD-10 diagnoses were classified as urgent or elective, categories into which the public/private surgery rates were also sorted.

**Results:**

The overall rate of orthopedic surgery was 7.54 per 1000 inhabitants in 2018. Patients covered under private insurance had an orthopedic surgery rate 2.23 times higher than patients within the public system (*p*-value < 0.001). This difference became more accentuated when sorting by elective surgeries, with private healthcare having a rate 2.97 times higher than public healthcare (*p*-value < 0.001). In the multivariate analysis, significant differences were observed in the rates of orthopedic surgery, being higher in the private system, elective surgeries, and older adults. No significant differences were observed according to sex (*p*-value 0.270).

**Conclusions:**

In Chile, access disparity to orthopedic surgical care existed between private and public healthcare systems, elective surgeries, and older age groups. Disparity in access became greater when separately analyzing the rates of elective and urgent orthopedic surgeries.

**Level of evidence:**

III.

**Supplementary Information:**

The online version contains supplementary material available at 10.1186/s12891-025-08295-7.

## Introduction

Disparity in healthcare access is a current subject of interest in medicine [1]. Health disparities have been defined as systematic, plausibly avoidable health differences adversely affecting socially disadvantaged groups [2]. When considering access to surgical care, these differences may directly influence the population’s morbidity, mortality, and the country’s global burden of disease [1, 3]. Studies investigating these differences may help understand the nature of these inequities and contribute to directing resource allocation policies.

Weiser et al. reported that worldwide access to surgical care is inequitably distributed across different countries, exposing the disproportionate scarcity of surgical access in low-income settings [3]. There is evidence of a wide number of factors that influence the population’s access to primary care, specialist referrals, and optimal treatment of their conditions [4–7].

In the field of orthopedic surgery, sex, race, geographic location, and socioeconomic status have established effects on the access to surgical care of selected groups [7–11]. Insurance status has also been shown to be determinant in the resolution of specific interventions such as lower extremity fractures and reconstructive and arthroscopic surgery, among others [7, 9, 12–16]. Insurance status may have a greater impact on the treatment of elective surgeries compared to urgent cases. This limits the opportunity to treat elective cases, in a scenario with limited resources. Despite the available literature, population-based studies remain lacking in regard to access to orthopedic surgery.

The aim of this study was to evaluate if access to orthopedic surgery differs by healthcare coverage in a country with a dual healthcare system adjusted by age, sex, and urgent and elective conditions. We hypothesized that differential access to orthopedic surgery would exist according to the type of healthcare coverage (i.e., private vs. public), having a greater impact on non-urgent conditions.

## Methods

### Design

A cross-sectional, population-based study investigated the rate of orthopedic surgery in Chile in 2018. The scientific ethics committee of the Pontifical Catholic University of Chile (ID Resolution 16–196) granted approval for this study in accordance with the Declaration of Helsinki and Chilean laws that regulate data management in research. To conduct this study, informed consent was not required from the participants, as the research was based on anonymous, publicly available national databases.

### Background

Chile is a developed country, and its sociodemographic data are described in Table 1 [17, 18]. Among members of the Organization for Economic Co-operation and Development (OECD), Chile has one of the highest levels of income inequality [19]. Healthcare is provided through two separate insurance systems – public and private [20]. In 2018, the distribution of beneficiaries by health insurance type was 75.2% in the public system, 18.0% in the private sector, and 6.8% in other categories, including police and armed forces [21].

**Table 1:** Chilean sociodemographic data for 2018

Population^a^	18,751,405
Life Expectancy^a^	80 years
Gross Domestic Product^b^ GINI Coefficient^b^	295.4 USD Billion 0,44

*Sources*: ^a^*National Institute of Statistics;* ^b^*World Bank* [17, 18]

Public healthcare insurance is funded through taxes paid by workers and pensioners in this system, corresponding to 7% of their income. It also covers unemployed individuals, dependent family members of insured workers, and the poor or indigent. However, enrollment in the public option is not mandatory, and individuals can choose to use the legally mandated 7% or more to purchase health insurance from multiple private companies.

Co-payments in the private sector are highly variable, disproportionately impacting lower socio-economic classes and those with greater health needs [22]. Consequently, beneficiaries of private insurance are more likely to access specialized medical services and experience a lower incidence of catastrophic spending compared to individuals relying on public insurance [19].

### Information sources

Demographic distribution of Chile’s population was extracted from the Chilean National Institute of Statistics [17]. Information regarding the number of beneficiaries covered by each type of healthcare insurance was obtained from the Chilean Ministry of Health [23].

The number of surgeries performed in 2018 was collected from the Hospital Discharge Registry (Department of Statistics, Chilean Ministry of Health). Information is mandatorily entered into this registry when patients are discharged from any healthcare center. This database is open access (https://deis.minsal.cl/) and provides patients’ demographic data, diagnosis, surgical procedures performed, and information about their healthcare insurance.

Musculoskeletal diagnoses were independently defined by two orthopedic surgeons from the ICD-10 (CIE-10) database. In cases of disagreement, a third orthopedic surgeon was consulted to reach a consensus. In all, 1,041 ICD-10 diagnoses were obtained from The National Discharge Registry from the Ministry of Health [13], the most frequent categories of which were M, Q, S, and T (Annex 1). Inclusion criteria considered patients operated and discharged with a musculoskeletal condition during the year 2018. This year was selected as the registry’s most recent full year when the database was obtained before the 2019 Chilean social upheaval and the 2020 COVID-19 pandemic. This year does not show significant differences from subsequent years in relation to health access and macroeconomic variables.

The ICD-10 diagnoses defined as musculoskeletal conditions were grouped according to the type of surgical treatment needed into elective (e.g., osteoarthritis) and urgent (e.g., fractures, acute infections), as per the aforementioned procedure.

### Statistical analysis

The annual rate of orthopedic surgeries per 1,000 inhabitants was calculated. Surgery access by healthcare insurance was compared by calculating the rate of surgeries in the private and public healthcare systems per 1,000 beneficiaries.

Rates of surgeries for each insurance type were compared using Negative Binomial regression to calculate the Incidence Rate Ratio (IRR). Multivariate regression was used to compare differences between health insurance (public versus private) adjusted by age (0–19 years; 20–44 years; 45–64 years; ≥65 years), sex, and surgery type (elective or urgent). A *p*-value of less than 0.05 was considered statistically significant.

Statistical analyses were performed with Stata LLC v.16 software (serial number 401606250896).

## Results

In 2018, Chile had 751,477 hospital discharges. Of these, 141,374 were orthopedic surgeries, accounting for 18.81% of all surgical procedures. The national rate of orthopedic surgeries was 7.54 per 1,000 inhabitants (CI 95% 7.50–7.58). Among these surgeries, male patients represented 56.69% (*n* = 77,977). The average age of patients was 46.37 years (standard deviation ± 20.69), while the median age was 48 years, with a range from 0 to 107 years.

In terms of healthcare insurance, the rate of orthopedic surgery in the public system was 5.77 per 1,000 inhabitants (CI95% 5.73–5.81), while the private system had a rate of 12.88 per 1,000 inhabitants (CI95% 12.76-13.00). When comparing both rates, the private system had an orthopedic surgery rate that was 2.23 times greater than that of the public system (CI 95% IRR 2.20–2.26; *p* < 0.001) (Table 2). Among the types of surgeries performed, 62,900 (44.49%) were urgent, while 78,474 (55.51%) were elective, with rates per 1,000 inhabitants of 3.35 and 4.18, respectively (Tables 3 and 4). The difference in surgery rates based on health insurance was significant, being 1.25 times greater in the private system compared to the public system (95% CI: IRR 1.23–1.26; *p* < 0.001) (Table 4). The difference between insurance types became accentuated when separating analyses based on elective and urgent surgeries. The annual rate of elective surgeries was 2.97 times greater in the private system (CI 95% 2.92–3.01; *p* < 0.001), while urgent surgeries in the private system occurred only at a rate 1.46 times more than in the public system (CI 95% 1.43–1.48; *p* < 0.001) (Table 3).

**Table 2:** Total and orthopedic (urgent/elective) surgeries performed in Chile in 2018, as well as respective rates

	*n*	Rate Per 1,000 Inhabitants^b^
Total Surgeries^a^	751,477	40.08 (39.99–40.16)
Orthopedic Surgeries^a^	141,374	7.54 (7.50–7.58)
Urgent	62,900	3.35 (3.33–3.38)
Elective	78,874	4.18 (4.16–4.21)

*Source*: ^a^*Ministry of Health*, ^b^*National Institute of Statistics*

**Table 3:** Annual rate of orthopedic surgeries, per 1,000 beneficiaries, by healthcare insurance for Chile in 2018

Type of Surgery	Public Insurance (CI 95%)	Private Insurance (CI 95%)	IRR^a^ (CI 95%)	*p*-value
Urgent	2.81 (2.79–2.84)	4.10 (4.03–4.17)	1.46 (1.43–1.48)	< 0.001
Elective	2.96 (2.93–2.99)	8.78 (8.68–8.87)	2.97 (2.92–3.01)	< 0.001
Total	5.77 (5.73–5.81)	12.88 (12.76-13.00)	2.23 (2.20–2.26)	< 0.001

*Univariate Negative Binomial Regression*

*Abbreviatures: CI: Confidence interval; IRR: Incidence rate ratio*

**Table 4:** Comparison of incidence orthopedic rate between groups

Variable	Number of surgeries	Population	Rate per 1000 habitants	IC95%		IRR	IC95%		*p*-value
**Health insurance**									
* Public*	82,218	14,242,655	5.77	5.73	5.81	*			
* Private*	43,844	3,404,896	12.88	12.76	13.00	2.23	2.20	2.26	< 0.001
**Type of surgery**									
* Urgent*	62,900	18,751,405	3.35	3.33	3.38	*			
* Elective*	78,474	18,751,405	4.18	4.16	4.21	1.25	1.23	1.26	< 0.001
**Sex**									
* Female*	63,397	9,506,921	6.67	6.62	6.72	*			
* Male*	77,977	9,244,484	8.43	8.38	8.49	1.26	1.25	1.28	< 0.001
**Age**									
* 0–19 years*	16,233	4,982,552	3.26	3.21	3.31	*			
* 20–44 years*	47,395	7,144,770	6.63	6.57	6.69	2.03	2.00	2.07	< 0.001
* 45–64 years*	49,081	4,458,888	11.00	10.91	11.10	3.38	3.32	3.44	0.002
* ≥ 65 years*	28,669	2,165,195	13.24	13.09	13.39	4.06	3.99	4.14	0.003

Negative binomial regression was used for the individual analysis of each variable. * reference variable

Regarding sex, the surgery rate for females was 6.67 per 1,000 inhabitants, while for males, it was 8.43, indicating a rate 1.26 times higher compared to females (95% CI: IRR 1.25–1.28; *p* < 0.001) (Table 4). Additionally, the age group showing the most significant differences in rates compared to those under 20 years old were older adults, with rates of 3.26 versus 13.24 per 1,000 inhabitants, resulting in a rate 4.06 times higher for older adults (95% CI: IRR 3.99–4.14; *p* < 0.003) (Table 4).

In the multivariate analysis, differences in orthopedic surgery rates remained statistically significant according to insurance type, surgery type, and age; however, those related to sex were not statistically significant (IRR 1.18, 95% CI: 0.88–1.58; *p* = 0.270) (Table 5). Overall, the annual rate of orthopedic surgery was 2.03 times greater in the private system (95% CI: 1.53–2.70; *p* < 0.001), and elective surgeries had a rate 1.43 times higher than urgent surgeries (95% CI: 1.07–1.92; *p* = 0.016) (Table 5).

**Table 5:** Comparison of incidence orthopedic rate between public and private health insurance, adjusted by type of surgery, sex and age

Variables	IRR	Std. Err.	CI 95%	*p*-value	
Health insurance					
* Public (reference)*					
* Private*	2.03	0.30	1.53–2.70	< 0.001	
Type of surgery					
* Elective (reference)*					
* Urgent*	1.43	0.21	1.07–1.92	0.016	
Sex					
* Female (reference)*					
* Male*	1.18	0.18	0.88–1.58	0.270	
Age					
* 0–19 years (reference)*					
* 20–44 years*	1.88	0.38	1.26–2.80	0.002	
* 45–64 years*	3.17	0.65	2.11–4.75	< 0.001	
* ≥ 65 years*	4.95	1.03	3.29–7.43	< 0.001	

*Multivariate Binomial Negative Regression.* *Abbreviature: IRR (incidence rate ratio); Std. Err (Standar Error); CI (Confidence Interval)*

## Discussion

This study evaluated the access to orthopedic surgery according to public and private health insurance in a country with a dual healthcare system. Our results confirmed the hypothesis that there is an important disparity in access to orthopedic surgical care. The population insured by private companies had a 2.23 times higher rate of surgeries than those under public insurance. In elective procedures, this difference is accentuated to 2.97 times higher in private insurance beneficiaries.

Recent literature has shown disparities in access to orthopedic care in specific populations. A large number of studies performed in the United States have compared the access to healthcare of groups under Medicaid and private insurance. These studies have found disparities in access to pediatric orthopedic care, differences in the time to surgery of patients with anterior cruciate ligament reconstruction surgery, and treatment of hand flexor tendon lacerations, among others [10, 12, 13, 16, 24, 25]. Despite the high prevalence of orthopedic surgery, there is limited evidence in Latin America regarding inequities in access. Vidal et al. observed that in Chile, the rotator cuff surgery rates were 3.4 times higher in the private healthcare system compared to the public system [7].

Our study suggests a large unaddressed orthopedic surgery burden in the public insured population, as this system covers 75% of Chile’s population [21]. Even though the aim of this study was not to analyze socioeconomic factors, the population that can opt for private insurances has higher levels of income [22]. This fact suggest that socioeconomic status may play a role in access to surgery in a dual healthcare system. The disparity found is represented mostly by limited access to elective surgeries. This can be explained by barriers in the public system which include limited access to primary care, delayed orthopedic surgeon referrals, and long waiting lists for surgery. Elective conditions are not a priority in a system with limited resources and a high demand to solve urgent cases. Additionally, the elevated rate of elective surgeries observed within the private system warrants careful analysis. It is important to consider that financial incentives may potentially impact surgical decision-making processes.

In our study, the group with the lowest rates of orthopedic surgery was the 0-19-year-old group (3.26 per 1,000 inhabitants), followed by the 20-44-year-old group (6.63 per 1,000 inhabitants). In contrast, the older adult group had a rate of 13.24, which was 4.06 times higher than that of individuals under 19 in the univariate analysis and 4.95 times higher in the multivariate analysis (both *p*-values < 0.001). These findings align with existing literature and can be attributed, in part, to demographic transition, characterized by a significant increase in the older adult population, whose comorbidities more frequently necessitate surgical intervention (e.g., hip fractures or osteoarthritis) [26–29]. For instance, a study conducted in Canada evaluating outpatient consultations found that the lowest percentage of surgical consultations was among those under 25 years of age, with 91% of consultations being non-surgical, compared to 75% of non-surgical consultations in older adults [30].

Regarding orthopedic surgery rates by sex, differences exist depending on the condition [31]. Males generally have a higher incidence of conditions such as shoulder injury [32, 33], while females have higher surgical rates of hip fractures and total joint arthroplasty [11, 34, 35]. In our study, we did not find significant differences in orthopedic surgery rates by sex in the multivariate analysis (IRR 1.18; *p*-value 0.270). This lack of significance may be attributed to our inclusion of all orthopedic diagnoses without analyzing specific pathologies where sex-related differences have been reported.

The main strength of this study is that it was a population study, being the first to assess access to orthopedic surgery for an entire country. A two-tier healthcare system was analyzed, which can be extrapolated to other countries with similar healthcare systems. The limitations of this study include a possible information bias. The analyzed data were obtained from national surveys and clinical forms, which are completed by individuals with different degrees of specializations and competencies. For this reason, there could be errors in coding of diagnoses and procedures. Furthermore, the selection of musculoskeletal ICD-10 diagnoses was arbitrary, as was subsequent classification into elective or urgent conditions. Classification of these diagnoses has not been standardized in the literature. More accurate statistics could be obtained by selecting cases based on surgical procedure codes. This information was not available in a standardized manner in our databases.

Our study confirms that there is an important disparity in access to orthopedic surgery in Chile according to healthcare insurance system. The limited access of the population under the public system was accentuated when analyzing elective surgeries. Future investigations should study the effect of other factors such as access to primary care physicians, orthopedic surgeon referral, waiting times for orthopedic surgery, and number of orthopedic surgery indications.

## Conclusion

Access to orthopedic surgical care differed between the private and public healthcare systems in Chile, according to the type of surgery and the patient’s age. The disparity in access became greater when the rates of elective and urgent orthopedic surgeries were separately analyzed.

## Electronic supplementary material

Below is the link to the electronic supplementary material.

Annex 1

Annex 2

## Data Availability

No datasets were generated or analysed during the current study.
