# Supplementary material for: Disparity in access to orthopedic surgery between public and private healthcare insurance: a nationwide population-based study
Source: BMC Musculoskelet Disord. 2025 May 10;26:458. doi: 10.1186/s12891-025-08295-7 (PMC12066059; doi:10.1186/s12891-025-08295-7)
Supplement: Supplementary file 1 — Annex 1 [file 12891_2025_8295_MOESM1_ESM.docx]

**Annex 1.** Diagnostics CIE-10 considerate to Elective Procedures Orthopedic Surgeries.

| A180 | B902 | B91 | C400 | C401 | C402 | C403 | C408 | C409 | C412 | C413 | C414 |
| --- | --- | --- | --- | --- | --- | --- | --- | --- | --- | --- | --- |
| C418 | C419 | C491 | C492 | C764 | C765 | D160 | D161 | D162 | D163 | D166 | D167 |
| D168 | D169 | D172 | D211 | D212 | D480 | G560 | G561 | G562 | G563 | G568 | G569 |
| G570 | G571 | G572 | G573 | G574 | G575 | G576 | G578 | G579 | M011 | M012 | M016 |
| M018 | M020 | M021 | M022 | M023 | M028 | M029 | M060 | M061 | M062 | M063 | M064 |
| M068 | M069 | M070 | M071 | M072 | M073 | M074 | M075 | M076 | M080 | M081 | M082 |
| M083 | M084 | M088 | M089 | M090 | M091 | M092 | M098 | M100 | M101 | M102 | M103 |
| M104 | M109 | M110 | M111 | M112 | M118 | M119 | M120 | M121 | M122 | M128 | M130 |
| M140 | M141 | M142 | M145 | M146 | M148 | M150 | M151 | M152 | M153 | M154 | M158 |
| M159 | M160 | M161 | M162 | M163 | M164 | M165 | M166 | M167 | M169 | M170 | M171 |
| M172 | M173 | M174 | M175 | M179 | M180 | M181 | M182 | M183 | M184 | M185 | M189 |
| M190 | M191 | M192 | M198 | M199 | M200 | M201 | M202 | M203 | M204 | M205 | M206 |
| M210 | M211 | M212 | M213 | M214 | M215 | M216 | M217 | M218 | M219 | M220 | M221 |
| M222 | M223 | M224 | M228 | M229 | M230 | M231 | M232 | M233 | M234 | M235 | M236 |
| M238 | M239 | M240 | M241 | M242 | M243 | M244 | M245 | M246 | M247 | M248 | M249 |
| M250 | M251 | M252 | M253 | M254 | M255 | M256 | M257 | M258 | M259 | M400 | M401 |
| M402 | M403 | M404 | M405 | M410 | M411 | M412 | M413 | M414 | M415 | M418 | M419 |
| M420 | M421 | M429 | M430 | M431 | M432 | M433 | M434 | M435 | M436 | M438 | M439 |
| M45X | M460 | M461 | M462 | M468 | M469 | M471 | M472 | M478 | M479 | M480 | M481 |
| M482 | M484 | M485 | M488 | M489 | M490 | M491 | M492 | M493 | M494 | M495 | M498 |
| M500 | M501 | M502 | M503 | M508 | M509 | M510 | M511 | M512 | M513 | M514 | M518 |
| M519 | M530 | M531 | M533 | M538 | M539 | M540 | M541 | M542 | M543 | M544 | M545 |
| M546 | M548 | M549 | M610 | M611 | M612 | M613 | M614 | M615 | M619 | M621 | M623 |
| M624 | M625 | M628 | M629 | M631 | M633 | M638 | M652 | M653 | M654 | M658 | M659 |
| M660 | M661 | M662 | M663 | M664 | M665 | M670 | M671 | M672 | M673 | M674 | M678 |
| M679 | M688 | M700 | M701 | M702 | M703 | M704 | M705 | M706 | M707 | M708 | M709 |
| M712 | M713 | M714 | M715 | M718 | M719 | M720 | M721 | M722 | M724 | M725 | M728 |
| M729 | M738 | M750 | M751 | M752 | M753 | M754 | M755 | M758 | M759 | M760 | M761 |
| M762 | M763 | M764 | M765 | M766 | M767 | M768 | M769 | M770 | M771 | M772 | M773 |
| M774 | M775 | M778 | M779 | M800 | M801 | M802 | M803 | M804 | M805 | M808 | M809 |
| M840 | M841 | M842 | M848 | M849 | M850 | M851 | M852 | M853 | M854 | M855 | M856 |
| M858 | M859 | M862 | M863 | M864 | M865 | M866 | M868 | M869 | M870 | M871 | M872 |
| M873 | M878 | M879 | M880 | M888 | M889 | M890 | M891 | M892 | M893 | M894 | M895 |
| M896 | M898 | M899 | M900 | M901 | M902 | M903 | M904 | M905 | M906 | M908 | M910 |
| M911 | M912 | M913 | M918 | M919 | M920 | M921 | M922 | M923 | M924 | M925 | M926 |
| M927 | M928 | M929 | M931 | M932 | M938 | M939 | M942 | M943 | M948 | M949 | M953 |
| M955 | M958 | M959 | M960 | M961 | M962 | M963 | M964 | M965 | M968 | M969 | M990 |
| M991 | M993 | M994 | M995 | M996 | M997 | M998 | M999 | Q650 | Q651 | Q652 | Q653 |
| Q654 | Q655 | Q656 | Q658 | Q659 | Q660 | Q661 | Q662 | Q663 | Q664 | Q665 | Q666 |
| Q667 | Q668 | Q669 | Q675 | Q680 | Q681 | Q682 | Q683 | Q684 | Q685 | Q688 | Q690 |
| Q691 | Q692 | Q699 | Q700 | Q701 | Q702 | Q703 | Q704 | Q709 | Q710 | Q711 | Q712 |
| Q713 | Q714 | Q715 | Q716 | Q718 | Q719 | Q720 | Q721 | Q722 | Q723 | Q724 | Q725 |
| Q726 | Q727 | Q728 | Q729 | Q730 | Q731 | Q738 | Q740 | Q741 | Q742 | Q743 | Q748 |
| Q749 | Q760 | Q761 | Q762 | Q763 | Q764 | Q765 | Q766 | Q767 | Q768 | Q769 | Q770 |
| Q771 | Q772 | Q773 | Q774 | Q775 | Q776 | Q777 | Q778 | Q779 | Q780 | Q781 | Q782 |
| Q783 | Q784 | Q785 | Q786 | Q788 | Q789 | S832 | S833 | S932 | T840 | T841 | T842 |
| T843 | T844 | T845 | T846 | T847 | T848 | T849 | T870 | T871 | T872 | T873 | T874 |
| T875 | T876 | T911 | T912 | T913 | T920 | T921 | T922 | T923 | T924 | T925 | T926 |
| T928 | T929 | T930 | T931 | T932 | T933 | T934 | T935 | T936 | T938 | T939 | Z946 |
| Z966 | Z967 |  |  |  |  |  |  |  |  |  |  |
