# Supplementary material for: Disparity in access to orthopedic surgery between public and private healthcare insurance: a nationwide population-based study
Source: BMC Musculoskelet Disord. 2025 May 10;26:458. doi: 10.1186/s12891-025-08295-7 (PMC12066059; doi:10.1186/s12891-025-08295-7)
Supplement: Supplementary file 2 — Annex 2 [file 12891_2025_8295_MOESM2_ESM.docx]

| **Annex 2.** Diagnostics CIE-10 considerate to Urgent Procedures Orthopedic Surgeries. | | | | | | | | | | | |
| --- | --- | --- | --- | --- | --- | --- | --- | --- | --- | --- | --- |
| M000 | M001 | M002 | M008 | M009 | M010 | M013 | M014 | M015 | M125 | M131 | M138 |
| M139 | M463 | M464 | M465 | M470 | M483 | M532 | M600 | M620 | M622 | M626 | M630 |
| M632 | M650 | M651 | M680 | M710 | M711 | M730 | M731 | M843 | M844 | M860 | M861 |
| M907 | M930 | M966 | M992 | S130 | S131 | S132 | S133 | S134 | S135 | S136 | S140 |
| S141 | S142 | S143 | S144 | S145 | S146 | S16 | S178 | S179 | S18 | S197 | S198 |
| S199 | S220 | S221 | S230 | S231 | S233 | S240 | S241 | S242 | S243 | S245 | S246 |
| S300 | S310 | S320 | S321 | S322 | S323 | S324 | S325 | S327 | S328 | S330 | S331 |
| S332 | S333 | S334 | S335 | S336 | S337 | S340 | S341 | S342 | S343 | S344 | S345 |
| S346 | S348 | S400 | S407 | S408 | S409 | S410 | S411 | S417 | S418 | S420 | S421 |
| S422 | S423 | S424 | S427 | S428 | S429 | S430 | S431 | S432 | S433 | S434 | S435 |
| S436 | S437 | S440 | S441 | S442 | S443 | S444 | S445 | S447 | S448 | S449 | S460 |
| S461 | S462 | S463 | S467 | S468 | S469 | S47 | S480 | S481 | S489 | S497 | S498 |
| S499 | S500 | S501 | S507 | S508 | S509 | S510 | S517 | S518 | S519 | S520 | S521 |
| S522 | S523 | S524 | S525 | S526 | S527 | S528 | S529 | S530 | S531 | S532 | S533 |
| S534 | S540 | S541 | S542 | S543 | S547 | S548 | S549 | S560 | S561 | S562 | S563 |
| S564 | S565 | S567 | S568 | S570 | S578 | S579 | S580 | S581 | S589 | S597 | S598 |
| S599 | S600 | S601 | S602 | S607 | S608 | S609 | S610 | S611 | S617 | S618 | S619 |
| S620 | S621 | S622 | S623 | S624 | S625 | S626 | S627 | S628 | S630 | S631 | S632 |
| S633 | S634 | S635 | S636 | S637 | S640 | S641 | S642 | S643 | S644 | S647 | S648 |
| S649 | S660 | S661 | S662 | S663 | S664 | S665 | S666 | S667 | S668 | S669 | S670 |
| S678 | S680 | S681 | S682 | S683 | S684 | S688 | S689 | S697 | S698 | S699 | S700 |
| S701 | S707 | S708 | S709 | S710 | S711 | S717 | S718 | S720 | S721 | S722 | S723 |
| S724 | S727 | S728 | S729 | S730 | S731 | S740 | S741 | S742 | S747 | S748 | S749 |
| S760 | S761 | S762 | S763 | S764 | S767 | S770 | S771 | S772 | S780 | S781 | S789 |
| S797 | S798 | S799 | S800 | S801 | S807 | S808 | S809 | S810 | S817 | S818 | S819 |
| S820 | S821 | S822 | S823 | S824 | S825 | S826 | S827 | S828 | S829 | S830 | S831 |
| S834 | S835 | S836 | S837 | S840 | S841 | S842 | S847 | S848 | S849 | S860 | S861 |
| S862 | S863 | S867 | S868 | S869 | S870 | S878 | S880 | S881 | S889 | S897 | S898 |
| S899 | S900 | S901 | S902 | S903 | S907 | S908 | S909 | S910 | S911 | S912 | S913 |
| S917 | S920 | S921 | S922 | S923 | S924 | S925 | S927 | S929 | S930 | S931 | S933 |
| S934 | S935 | S936 | S940 | S941 | S942 | S943 | S947 | S948 | S949 | S960 | S961 |
| S962 | S967 | S968 | S969 | S970 | S971 | S978 | S980 | S981 | S982 | S983 | S984 |
| S997 | S998 | S999 | T002 | T003 | T006 | T012 | T013 | T016 | T020 | T021 | T022 |
| T023 | T024 | T025 | T026 | T027 | T028 | T029 | T030 | T031 | T032 | T033 | T034 |
| T038 | T039 | T040 | T041 | T042 | T043 | T044 | T047 | T048 | T049 | T050 | T051 |
| T052 | T053 | T054 | T055 | T056 | T058 | T059 | T064 | T08 | T10 | T110 | T111 |
| T112 | T113 | T114 | T115 | T116 | T118 | T119 | T12 | T130 | T131 | T132 | T133 |
| T134 | T135 | T136 | T138 | T139 | T793 | T796 |  |  |  |  |  |
